# The impact of high-risk medications on mortality risk among older adults with polypharmacy: evidence from the English Longitudinal Study of Ageing

**DOI:** 10.1186/s12916-021-02192-1

**Published:** 2021-12-16

**Authors:** Yun-Ting Huang, Andrew Steptoe, Li Wei, Paola Zaninotto

**Affiliations:** 1 Department of Epidemiology and Public Health, University College London, 1-19 Torrington Place, London, WC1E 7HB UK; 2 Department of Behavioural Science and Health, University College London, London, UK; 3 School of Pharmacy, University College London, London, UK

**Keywords:** Ageing, Older people, High-risk medications, Mental health drugs, Opioids, Muscle relaxants, Polypharmacy, All-cause mortality, Cardiovascular disease mortality, Pharmacoepidemiology

## Abstract

**Background:**

Polypharmacy is common among older people and is associated with an increased mortality risk. However, little is known about whether the mortality risk is related to specific medications among older adults with polypharmacy. This study therefore aimed to investigate associations between high-risk medications and all-cause and cause-specific mortality among older adults with polypharmacy.

**Methods:**

This study included 1356 older adults with polypharmacy (5+ long-term medications a day for conditions or symptoms) from Wave 6 (2012/2013) of the English Longitudinal Study of Ageing. First, using the agglomerative hierarchical clustering method, participants were grouped according to the use of 14 high-risk medication categories. Next, the relationship between the high-risk medication patterns and all-cause and cause-specific mortality (followed up to April 2018) was examined. All-cause mortality was assessed by Cox proportional hazards model and competing-risk regression was employed for cause-specific mortality.

**Results:**

Five high-risk medication patterns—a renin-angiotensin-aldosterone system (RAAS) inhibitors cluster, a mental health drugs cluster, a central nervous system (CNS) drugs cluster, a RAAS inhibitors and antithrombotics cluster, and an antithrombotics cluster—were identified. The mental health drugs cluster showed increased risks of all-cause (HR = 1.55, 95%CI = 1.05, 2.28) and cardiovascular disease (CVD) (SHR = 2.11, 95%CI = 1.10, 4.05) mortality compared with the CNS drug cluster over 6 years, while others showed no differences in mortality. Among these patterns, the mental health drugs cluster showed the highest prevalence of antidepressants (64.1%), benzodiazepines (10.4%), antipsychotics (2.4%), antimanic agents (0.7%), opioids (33.2%), and muscle relaxants (21.5%). The findings suggested that older adults with polypharmacy who took mental health drugs (primarily antidepressants), opioids, and muscle relaxants were at higher risk of all-cause and CVD mortality, compared with those who did not take these types of medications.

**Conclusions:**

This study supports the inclusion of opioids in the current guidance on structured medication reviews, but it also suggests that older adults with polypharmacy who take psychotropic medications and muscle relaxants are prone to adverse outcomes and therefore may need more attention. The reinforcement of structured medication reviews would contribute to early intervention in medication use which may consequently reduce medication-related problems and bring clinical benefits to older adults with polypharmacy.

**Supplementary Information:**

The online version contains supplementary material available at 10.1186/s12916-021-02192-1.

## Background

Polypharmacy is a justifiable result of multimorbidity and has become prevalent among older people. Polypharmacy is commonly defined as taking five or more medications concurrently in the literature, although there is no agreed definition of polypharmacy [1]. Beyond the numerical definition, a concept of appropriate or problematic polypharmacy has been advocated by the National Institute for Health and Care Excellence (NICE) [2] and National Health Service (NHS) England [3]. In response to the fact that polypharmacy is related to under- or over-prescribing [4], appropriate polypharmacy refers to prescribed medications being optimised with the best evidence. Nevertheless, clinical guidelines are single-disease-based, which may not take the complexity of multimorbidity and polypharmacy into account, and the evidence for the optimisation of non-prescription medications seems to be unavailable. Furthermore, the assessment of polypharmacy is subject to data availability in population-based studies and must be individualised for each person.

There is evidence of an association between polypharmacy and all-cause mortality [5–11], in which polypharmacy is in part an indicator for the burden of diseases (e.g. disease severity). Dose-response relationships between deferent levels of polypharmacy and all-cause and CVD mortality among older adults were also found in our previous work [12]. However, little is known about which medication combinations within polypharmacy further relate to mortality. To date, a small number of studies have investigated the associations between medication use and all-cause mortality, but not in the context of polypharmacy, which is the focus of this study. Results from these studies are mixed. Anticholinergic medications (assessed using various scales) [13–15], opioids, antihistamines, and psychotropics [16] were reportedly related to an increased risk of all-cause mortality, whereas skeletal muscle relaxants showed lower risk, compared with not using muscle relaxants [16]. Another study investigated the associations between the use of 20 common drug classes and 1-year mortality among older people, according to hospitalisation status [17]. Several drug classes (e.g. lipid-lowering agents, calcium channel blockers (CCBs), and anxiolytics) were associated with reduced mortality, whereas some medications showed higher death rates (e.g. loop diuretics, digitalis and antiarrhythmic agents). Some medications, however, such as angiotensin-converting enzyme inhibitors (ACEIs), showed inconsistent results between hospitalised and non-hospitalised samples.

In addition to the medication categories that have been reported to be related to higher or lower mortality, some medications are believed to have a high probability of adverse effects among older adults, such as opioids, benzodiazepines (BZDs), and antihypertensive drugs [16, 18–20]. The ageing process is typically accompanied by changes in pharmacokinetics (absorption, distribution, metabolism and elimination) and pharmacodynamics, resulting in a more unpredictable performance of medications in older adults. There have been different strategies for the management of polypharmacy in clinical practice, advocated by different organisations across countries [21]. In the UK, the medication review is specifically targeted at polypharmacy in the NICE guidelines [22] and at heightened polypharmacy (10 or more medications) in the NHS England guidelines [3] and Scottish government guidance [23]. Apart from the concept of polypharmacy, people on high numbers of addictive pain management medications and those on high-risk medications are advised to have a medication review according to NHS England [3] and the Scottish government [23], respectively. Compared with NICE and NHS England, the Scottish government has set up extensive polypharmacy guidance that targets people on high-risk medications, regardless of the number of drugs taken [23]. High-risk medications are defined by 17 case-finding indicators, denoting the use of specific medications is linked to a high risk of specific symptoms or conditions. Some examples are the concurrent use of oral anticoagulant and antiplatelet linked to bleeding, prescribed methotrexate without folic acid linked to bone marrow suppression and high-dose opioids (equivalent to > 180 mg morphine per day) over the last 6 months linked to opioid dependency [23]. On the other hand, it seems that guidance on polypharmacy management outside the UK—Australia [24], Germany [25], and USA [26]—puts more emphasis on the utilisation of Beers criteria [27], the Screening Tool of Older People’s Prescriptions (known as STOPP), Screening Tool to Alert to Right Treatment (known as START) criteria [28], and the Medication Appropriateness Index criteria [29] to identify inappropriate prescribing. Moreover, Canada provides separate deprescribing guidelines and algorithms for certain medication categories, including proton pump inhibitors, antihyperglycemic agents, antipsychotics, BZD receptor agonists, and cholinesterase inhibitors and memantine [30].

To summarise, there has been little research into the types of medication use within polypharmacy in observational studies of nationally representative samples of older adults. Despite the finding that polypharmacy is associated with increased mortality, little is known about whether high-risk medications (either singly or in combined use) contribute to added risk among older people with polypharmacy. Also, there are disparities in the inclusion of high-risk medications in different guidelines. Thus, this study aimed to investigate the effect of high-risk medications on all-cause and cause-specific mortality in a nationally representative sample of older adults with polypharmacy. It was hypothesised that specific high-risk medications (e.g. anticholinergic agents or opioids) might increase the risk of mortality in older adults with polypharmacy.

## Methods

### Study population

The data came from Wave 6 (2012 − 2013) of the English Longitudinal Study of Ageing (ELSA), a nationally representative study of adults in England age 50 and older living in private households [31, 32]. Data collection is carried out every 2 years using computer-assisted interviews followed by self-completion questionnaires, and every 4 years through home visits from a study nurse during which biological samples and anthropometric measurements are taken [33, 34]. In Wave 6, a total of 9169 interviews with core members were conducted. Of these, 7730 participants were visited by a study nurse who recorded information on all medications. After the exclusion of people who had cancer (self-reported diagnoses or relevant treatments) (*N* = 499), 1705 participants with polypharmacy were involved in the cluster analysis. Polypharmacy was defined as taking five or more long-term medications for conditions or symptoms per day (Additional file 1: Table S1). After the cluster analysis was carried out, participants who did not have complete information on variables in the model (*N* = 328) (Additional file 1: Table S2) and those who had died within 1 year of follow-up (*N* = 21) were further excluded, resulting in an analytical sample of 1356 individuals with polypharmacy for the survival analysis (Additional file 1: Figure S1).

### High-risk medications

The medication data was collected by study nurses during home visits. By protocol, study nurses asked participants to show the containers for all the medications currently being taken and recorded them. High-risk medications for older people were identified from the literature [16, 18–20] on the grounds that they had a high probability of adverse effects in the ageing population. These high-risk medications were classified into 14 medication categories according to their pharmacological mechanisms (Additional file 1: Table S3), and they were subsequently employed in cluster analysis to group participants into a set of clusters. The 14 medication categories were BZDs, antipsychotics, antidepressants, antimanic agents, CCBs, diuretics, RAAS inhibitors, opioids, muscle relaxants, non-steroidal anti-inflammatory drugs (NSAIDs), antithrombotics, steroids, anticholinergics, and other CNS drugs. All medication categories were binary variables that denoted whether the participant was taking the medication or not.

### Mortality data

Study participants were linked to the NHS’s Central Register. For each deceased participant up to the end of follow-up (April 2018), the month and year of death were recorded. For participants with no record of an event, the data was censored at the end of May 2018. The causes of death were classified based on the International Classification of Diseases and categorised into CVD (codes I00–I99) and non-CVD, including cancer (codes C00–C97), diseases of the respiratory system (codes J00–J99), and other remaining causes.

### Potential confounders

This study included factors that had been reported in the literature or shown to be significantly related to the outcome in the univariable analysis. The socio-demographic characteristics were a continuous variable of age (years), binary variables of gender (male and female) and cohabiting status (living or not with a partner), and a categorical variable of total wealth (quintiles). Health factors included six long-term conditions (diabetes mellitus, coronary heart disease (CHD), stroke, lung disease (including asthma), Parkinson’s disease, and dementia (including Alzheimer’s disease)), an illness count of the remaining conditions (e.g. hypertension and psychiatric conditions), functional impairment (difficulty in either activities of daily living (ADLs) or instrumental activities of daily living (IADLs)), mobility difficulty, obesity (high body mass index (BMI) and waist circumference, and either high BMI or high waist circumference), smoking status (i.e. whether a current smoker or not), sleep duration (7 to 9 h, versus less than 7 or over 9 h), low physical activity, cognitive function (scores of zero to 20 on a set of tests of memory, including immediate and 5-min delayed recall), and significant depressive symptoms (four or more symptoms on the eight-item version of the Centre for Epidemiological Studies Depression Scale). The six long-term conditions were assessed individually because they have been identified as specific risk factors for mortality in the literature [7, 10, 11]. The self-reported long-term conditions were verified by medication profiles wherever possible, and three diagnoses—hyperuricemia (including gout), epilepsy, and inflammatory bowel disease—were identified by recognisably specific treatments (Additional file 1: Table S4).

### Statistical analysis

#### Cluster analysis

An agglomerative hierarchical clustering approach, with Ward’s linkage and the simple matching coefficient, was employed to group participants by taking account of similarity among the 14 medication categories [35–37], as in a previous study that grouped participants according to conditions [38]. In light of the study sample size (*N* = 1705), a maximum of 10 clusters was advisable in order to obtain sufficient statistical power in each cluster (a dendrogram is shown in Additional file 1: Figure S2). In cases where the clusters showed any differences in mortality and clear medication patterns, fewer clusters were chosen to allow each cluster to have a large enough sample to ensure a higher statistical power. The results indicated that five clusters (named clusters 1 − 5) fitted the data the best (other solutions to the clusters are presented in Additional file 1: Table S5). The clusters were labelled based on the most prevalent medication within each cluster (80% or more); if this was not applicable, the labelling was based on the medication category with the highest prevalence across the five clusters.

#### Survival analysis

Five clusters (clusters 1 − 5) with sample sizes of 194, 298, 387, 352, and 125, respectively, were employed in the survival analysis (Additional file 1: Figure S1). The potential confounders were entered into the model simultaneously. The association between the five clusters (medication patterns) and all-cause mortality was assessed by Cox proportional hazards regression. The hazard ratios (HRs) and corresponding 95% confidence intervals (CIs) from the Cox regression and the cumulative hazard functions of clusters (medication patterns) were presented. A competing-risk regression based on Fine and Gray’s proportional subhazard model [39] was used to analyse cause-specific mortality, as it takes account of competing events that prevent the event of interest from occurring. The subdistribution hazard ratios (SHRs) and corresponding 95% CIs from the competing-risk regression and the cumulative incidence function (CIF) denoting estimations of the incidence of the event were reported. The proportionality of hazards and subhazards was tested by using Schoenfeld residuals [40, 41], and no violation of assumptions was observed. The multicollinearity among the variables in our model was tested and ruled out. The cluster (medication pattern) with the lowest mortality and/or largest sample size was used as the reference group. Power calculations for survival analysis were provided in Additional file 1: Table S6. Statistical analyses were conducted using Stata (version 15.1; StataCorp LP, College Station, TX, USA).

#### Sensitivity analyses

Several sensitivity analyses were performed to ensure the robustness of the main findings. The first sensitivity analysis (SA1) included four cardiovascular-related diseases (i.e. CHD, stroke, hypertension, and other heart problems) individually rather than combining some of them into an illness count. The second sensitivity analysis (SA2) separated the diagnosis of psychiatric conditions from the illness count and adjusted for them individually. In the third sensitivity analysis (SA3), alcohol consumption was added as a covariate to the main model; this was not included in the primary analyses because of the reduced sample size with alcohol data (*N* = 1221). The fourth sensitivity analysis (SA4) included as an adjustment variable an indicator of inconsistency between medication use and self-reported conditions, to check whether the results were sensitive to the fact that some participants took cardiovascular or lipid-lowering medications but did not report relevant diagnoses. Lastly, to assess any potential bias due to missing data on the variables of interest, a supplementary analysis was carried out to examine baseline characteristics of participants between the sample with missing data (*N* = 1705) and complete cases (*N* = 1356).

## Results

### Prevalence of medication categories

The prevalence of 14 high-risk medication categories is shown in Table 1. RAAS inhibitors showed the highest prevalence (62.0%), followed by antithrombotics (56.8%), diuretics (39.3%), CCBs (37.6%), and antidepressants (22.6%). Opioids (12.9%), NSAIDs (11.8%), and other CNS drugs (10.0%) revealed similar prevalence rates among people with polypharmacy. The prevalence of the remaining medication groups was less than 10%.

**Table 1:** Prevalence of 14 high-risk medication categories in people with polypharmacy (*N* = 1356), ELSA 2012

Medication category	% (***N***)
RAAS inhibitors	62.0 (841)
Antithrombotics	56.8 (770)
Diuretics	39.3 (533)
CCBs	37.6 (510)
Antidepressants	22.6 (307)
Opioids for pain relief	12.9 (175)
NSAIDs^b^	11.8 (160)
Other CNS drugs	10.0 (136)
Muscle relaxants	6.1 (82)
Steroids^b^	6.0 (81)
BZDs^a^	5.6 (76)
Anticholinergics^c^	5.5 (75)
Antipsychotics	1.1 (15)
Antimanic agents	0.3 (4)

^a^Including sedatives

^b^Oral form only

^c^Remaining anticholinergics not included in other medication categories

### Medication pattern clusters

**Fig. 1:**
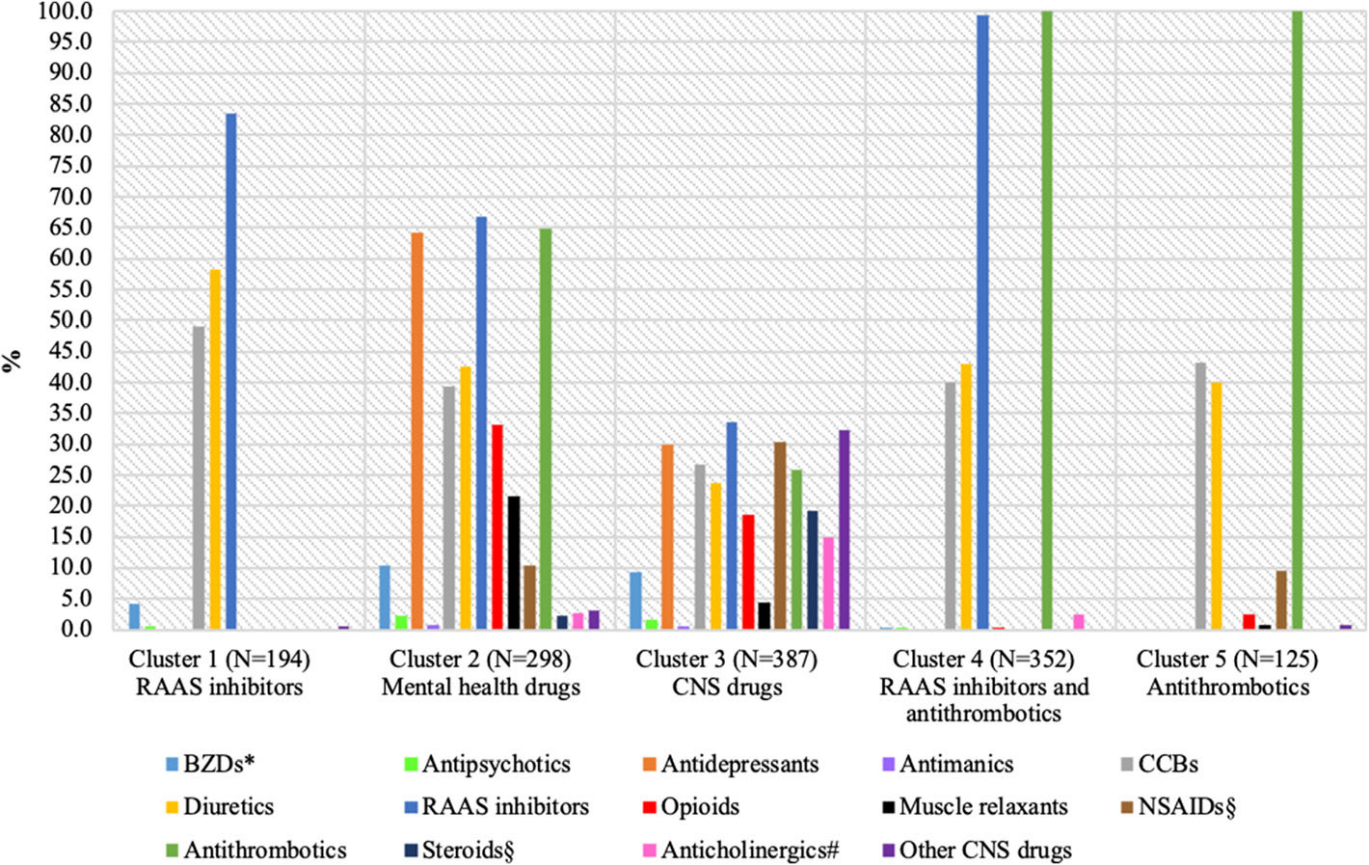
Prevalence of 14 high-risk medication categories across clusters, ELSA 2012. ^*^Including sedatives. ^§^Oral form only. ^#^Remaining anticholinergics not included in other medication categories

Based on the 14 high-risk medication categories, five clusters (medication patterns) were identified among people with polypharmacy. The distribution of medication categories across the five clusters is displayed in Fig. 1.

- Cluster 1 consisted of 194 participants who were frequent users of RAAS inhibitors (83.5%), diuretics (58.3%), and CCBs (49.0%), so cluster 1 was labelled ‘RAAS inhibitors’.
- Cluster 2 comprised 298 individuals, of whom over half took RAAS inhibitors (66.8%), antithrombotics (64.8%), and antidepressants (64.1%). This cluster also had the highest prevalence of BZDs (10.4%), antipsychotics (2.4%), and antimanic medications (0.7%), and it therefore was labelled ‘mental health drugs’.
- Cluster 3 consisted of 387 people who did not demonstrate a clear trend in the use of any specific medications. Only four medication categories had a prevalence of 30% or more: RAAS inhibitors (33.6%), other CNS drugs (32.3%), NSAIDs (30.2%), and antidepressants (30.0%). This cluster was labelled ‘CNS drugs’ because it had the highest prevalence of other CNS drugs compared with other clusters.
- Cluster 4 comprised 352 individuals who made combined use of RAAS inhibitors and antithrombotics (99.4 and 100.0%). Approximately 40% of these participants were on diuretics and CCBs, while only a few took any of the remaining medication categories. As a result, this cluster was labelled ‘RAAS inhibitors and antithrombotics’.
- Cluster 5 consisted of 125 users of antithrombotics (100.0%), of whom 43.2% used CCBs and 40.0% used diuretics. It was therefore labelled ‘antithrombotics’.

Among the five clusters, three medication patterns—the RAAS inhibitors cluster, the antithrombotics cluster, and the RAAS inhibitors and antithrombotics cluster—were more cardiovascular-oriented, since four medication categories—CCBs, diuretics, RAAS inhibitors, and antithrombotics—were mainly involved. On the other hand, the mental health drugs cluster and the CNS drugs cluster showed a broad spectrum of medication groups, although some had a low prevalence. The mental health drugs cluster showed higher prevalence rates than the CNS drug cluster in medications for mental illness (e.g. antidepressants, 64.1% versus 30.0%), cardiovascular medications (e.g. RAAS inhibitors, 66.8% versus 33.6%), opioids (33.2% versus 18.6%), and muscle relaxants (21.5% versus 4.4%). In contrast, the CNS drugs cluster had higher proportions of NSAIDs (30.2%), steroids (19.1%), anticholinergics (15.0%), and other CNS drugs (32.3%) than the mental health drugs cluster.

### Sample characteristics across medication patterns

The baseline characteristics of these participants according to the five medication patterns are summarised in Table 2. For simplicity, results for four variables that had similar proportions across the five clusters (total wealth and cognitive function) or low prevalence (Parkinson’s disease and dementia) are presented in Additional file 1: Table S7. The antithrombotics cluster was characterised by people with the highest average age (mean age 75.2 years), and the CNS drugs cluster had the highest proportion of women (63.6%). Different clusters showed the highest prevalence of different long-term conditions: diabetes (46.9%) in the RAAS inhibitors cluster, CHD (43.5%) in the RAAS inhibitors and antithrombotics cluster, stroke (20.1%) in the mental health drugs cluster, lung disease (43.4%) in the CNS drugs cluster, and the largest number of remaining conditions (median four) in the mental health drugs cluster. Both the mental health drugs cluster and the CNS drugs cluster revealed a higher prevalence of functional impairment (52.0% versus 52.5%), mobility difficulty (88.9% versus 83.5%), current smokers (16.8% versus 16.5%), low physical activity (48.0% versus 42.9%), and significant depressive symptoms (30.2% versus 24.6%) than the other clusters.

**Table 2:** Baseline characteristics^a^ of people with polypharmacy (*N* = 1356) by cluster, ELSA 2012

	Cluster 1 RAAS inhibitors	Cluster 2 Mental health drugs	Cluster 3 CNS drugs	Cluster 4 RAAS inhibitors + antithrombotics	Cluster 5 Antithrombotics
	(***N*** = 194) % (***N***)	(***N*** = 298) % (***N***)	(***N*** = 387) % (***N***)	(***N*** = 352) % (***N***)	(***N*** = 125) % (***N***)
Age (years) mean (SD)	70.9 (8.2)	71.3 (9.4)	70.3 (8.5)	73.4 (8.2)	75.2 (8.3)
Women	53.6 (104)	61.1 (182)	63.6 (246)	41.8 (147)	52.8 (66)
Living with a partner	71.1 (138)	56.7 (169)	64.9 (251)	63.9 (225)	52.8 (66)
Diabetes mellitus	46.9 (91)	37.9 (113)	25.1 (97)	37.2 (131)	32.8 (41)
CHD	12.9 (25)	38.3 (114)	14.2 (55)	43.5 (153)	39.2 (49)
Stroke	4.1 (8)	20.1 (60)	7.0 (27)	14.8 (52)	15.2 (19)
Lung disease (including asthma)	33.5 (65)	28.2 (84)	43.4 (168)	20.7 (73)	26.4 (33)
Number of conditions^b^ median (IQR)	3 (2)	4 (2)	3 (2)	3 (2)	3 (2)
Functional impairment^c^	26.8 (52)	52.0 (155)	52.5 (203)	28.7 (101)	29.6 (37)
Mobility difficulty^d^	68.6 (133)	88.9 (265)	83.5 (323)	71.6 (252)	84.0 (105)
Obesity					
High BMI and waist circumference	50.5 (98)	49.3 (147)	40.1 (155)	38.4 (135)	40.0 (50)
Either high BMI or high waist circumference	26.8 (52)	25.2 (75)	24.0 (93)	27.3 (96)	28.0 (35)
Current smoker	11.3 (22)	16.8 (50)	16.5 (64)	9.4 (33)	5.6 (7)
Sleep < 7 or > 9 h	38.7 (75)	46.6 (139)	53.8 (208)	39.2 (138)	49.6 (62)
Low physical activity	29.4 (57)	48.0 (143)	42.9 (166)	31.8 (112)	38.4 (48)
Depressive symptoms 4+	7.7 (15)	30.2 (90)	24.6 (95)	11.9 (42)	11.2 (14)

^a^All characteristics showed significantly different proportions among the five clusters. Two variables (total wealth and cognitive function) with similar distributions across the five clusters and two conditions (Parkinson’s disease and dementia (including Alzheimer’s disease)) with low prevalence rates are shown in Additional file 1: Table S7

^b^The remaining other conditions, not including diabetes mellitus, CHD, lung disease, Parkinson’s disease, and dementia (including Alzheimer’s disease)

^c^Defined as any difficulty in either ADLs or IADLs

^d^Defined as any difficulty in the movement of the arms or lower limbs

The information on all-cause and cause-specific mortality for the five medication patterns is summarised in Table 3. The smallest percentage of all-cause (12.9%) and CVD mortality (4.1%) was observed in the CNS drugs cluster, but the lowest non-CVD mortality (8.2%) appeared in the RAAS inhibitors and antithrombotics cluster. By contrast, the highest prevalence of all-cause (24.0%) and non-CVD mortality (16.0%) was detected in the antithrombotics cluster, while the mental health drugs cluster showed the highest CVD mortality (10.4%). The CNS drugs cluster was therefore treated as the reference group, because it had the largest sample size and the lowest all-cause and CVD mortality.

**Table 3:** Mortality^a^ in people with polypharmacy (*N* = 1356) by cluster, ELSA 2018

	Cluster 1 RAAS inhibitors	Cluster 2 Mental health drugs	Cluster 3 CNS drugs	Cluster 4 RAAS inhibitors + antithrombotics	Cluster 5 Antithrombotics
	(***N*** = 194) % (***N***)	(***N*** = 298) % (***N***)	(***N*** = 387) % (***N***)	(***N*** = 352) % (***N***)	(***N*** = 125) % (***N***)
All-cause mortality	15.5 (30)	22.2 (66)	12.9 (50)	16.8 (59)	24.0 (30)
CVD mortality	4.6 (9)	10.4 (31)	4.1 (16)	8.5 (30)	8.0 (10)
Non-CVD mortality	10.8 (21)	11.7 (35)	8.8 (34)	8.2 (29)	16.0 (20)

^a^Data was collected before May 2018

### The impact of medication patterns on mortality

**Fig. 2:**
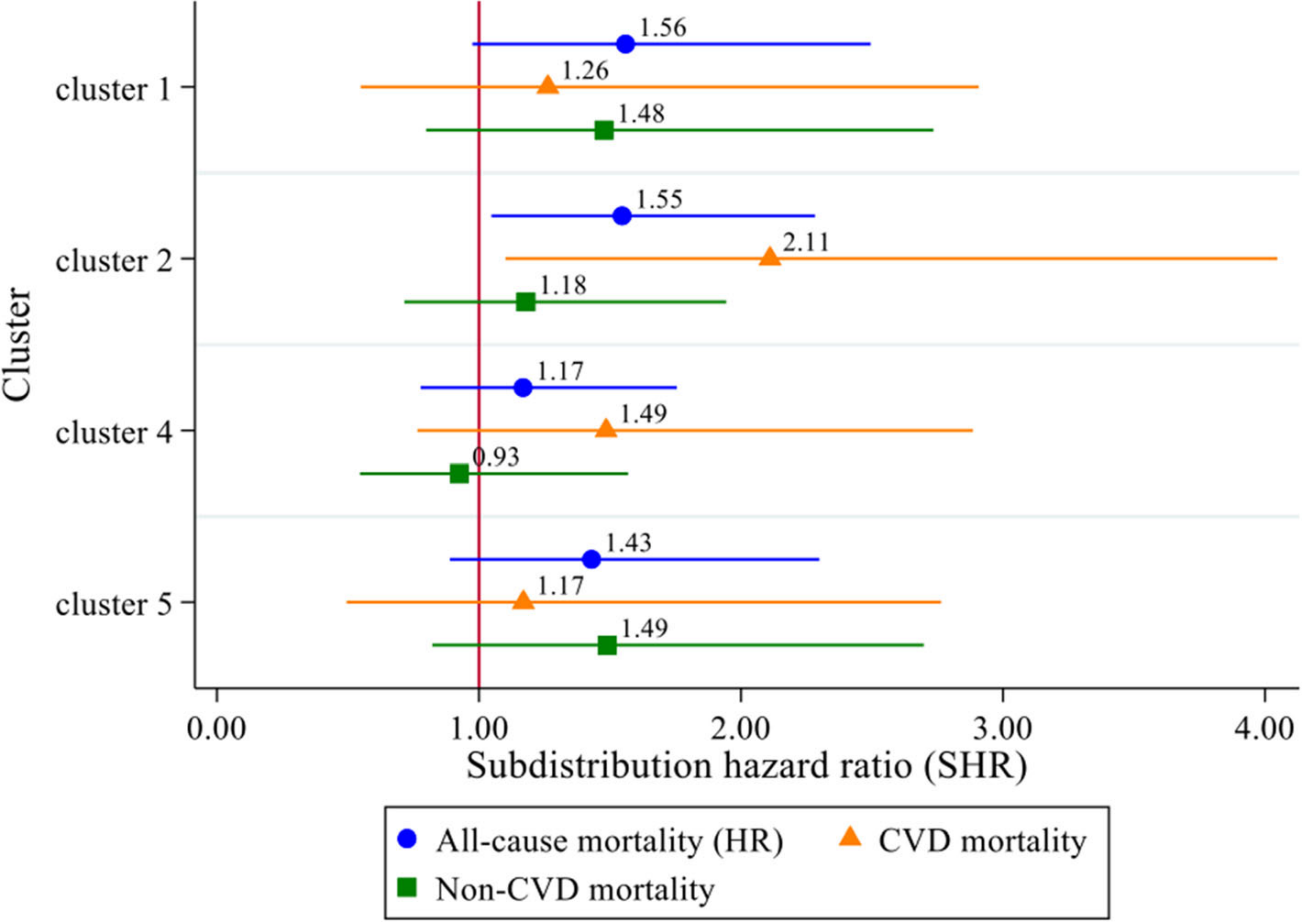
Associations^#^ between medication patterns^*^ and mortality in England in 2012 − 2018. ^#^Adjusted for age, gender, cohabitation, wealth, six long-term conditions (diabetes, CHD, stroke, lung disease, Parkinson’s disease, and dementia (including Alzheimer’s disease)), an illness count of the remaining conditions, functional impairment, mobility difficulty, obesity, smoking status, sleep duration, low physical activity, cognitive function, and depressive symptoms. ^*^Cluster 1 = RAAS inhibitors cluster; cluster 2 = mental health drugs cluster; cluster 3 = CNS drugs cluster (reference); cluster 4 = RAAS inhibitors and antithrombotics cluster; cluster 5 = antithrombotics cluster

**Fig. 3:**
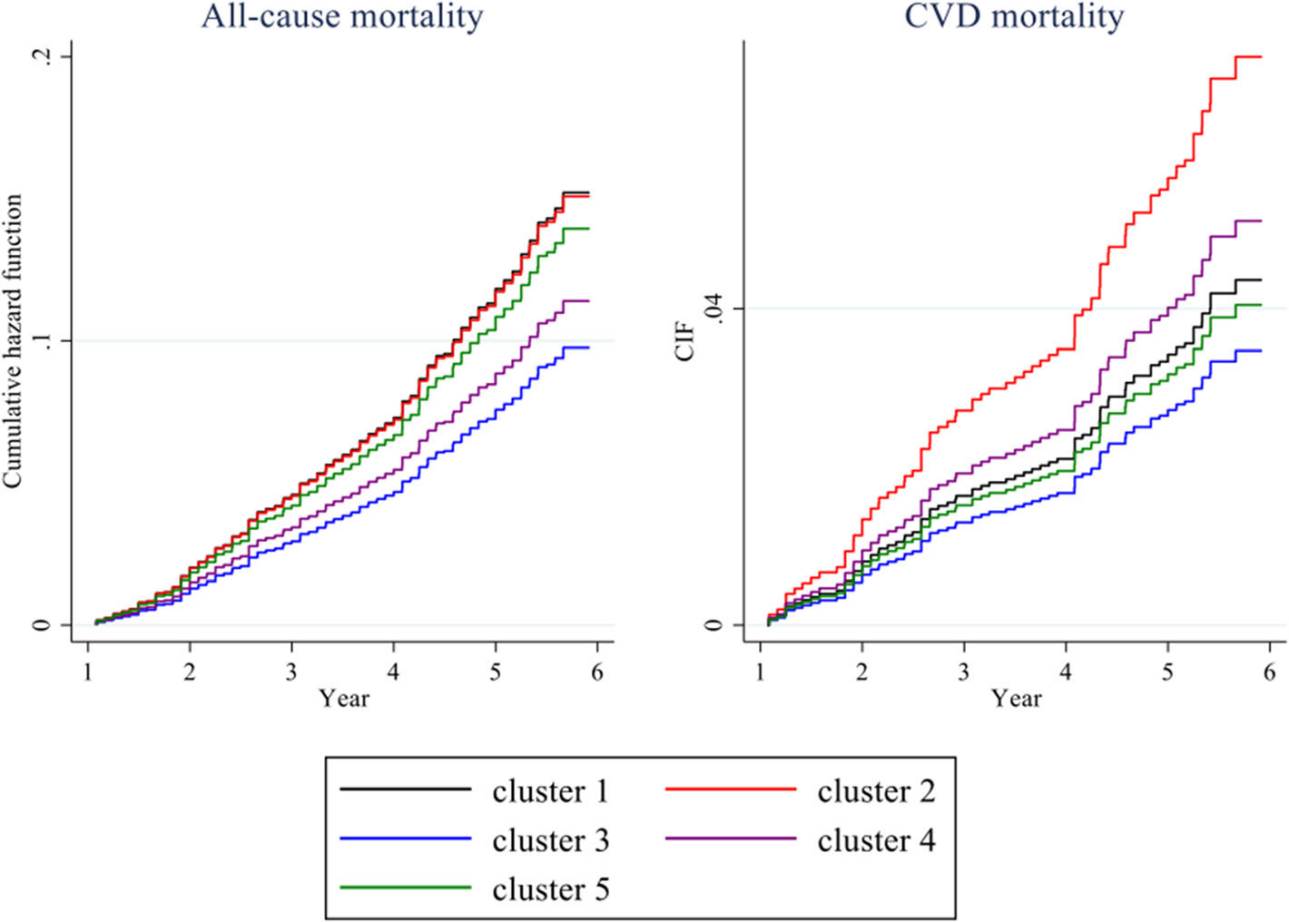
Cumulative hazard function of all-cause mortality and CIF of CVD mortality for medication patterns* in England in 2012 − 2018. ^*^Cluster 1 = RAAS inhibitors cluster; cluster 2 = mental health drugs cluster; cluster 3 = CNS drugs cluster (reference); cluster 4 = RAAS inhibitors and antithrombotics cluster; cluster 5 = antithrombotics cluster

Figure 2 shows the results of the association between medication patterns (at baseline in 2012) and mortality (up to 2018). Over the 6-year follow-up, only the mental health drugs cluster (cluster 2) showed a raised risk of all-cause mortality (HR = 1.55, 95% CI = 1.05, 2.28, *p* = 0.028) and CVD mortality (SHR = 2.11, 95% CI = 1.10, 4.05, *p* = 0.024) compared with the CNS drugs cluster (cluster 3). Neither the RAAS inhibitors cluster (cluster 1), the RAAS inhibitors and antithrombotics cluster (cluster 4), nor the antithrombotics cluster (cluster 5) revealed any differences in all-cause, CVD, or non-CVD mortality. The cumulative hazard function of all-cause mortality and the CIF of CVD mortality for the five medication patterns are presented in Fig. 3.

### Sensitivity analyses

The results of the sensitivity analyses are summarised in Additional file 1: Table S8. SA1 individually adjusted for each cardiovascular-related diagnosis—i.e. CHD, stroke, hypertension, and other heart problems—and obtained similar findings to the primary analysis (the mental health drugs cluster: HR = 1.53 versus 1.55 for all-cause mortality, SHR = 2.10 versus 2.11 for CVD mortality). SA2 adjusted for psychiatric conditions separately, and the results remained the same (the mental health drugs cluster: HR = 1.54 for all-cause mortality, SHR = 2.13 for CVD mortality). SA3 additionally included alcohol consumption, with a reduced sample size (*N* = 1221). The associations were unchanged, although the estimates of the risk of mortality for the mental health drugs cluster were slightly higher than those found in the primary analysis, with 1.70 versus 1.55 for all-cause mortality, and 3.04 versus 2.11 for CVD mortality. In SA4, an indicator of inconsistency between medication use and self-reported conditions was added to the main model. This indicator aimed to adjust for long-term conditions as comprehensively as possible by taking account of people on cardiovascular or lipid-lowering medications but without relevant diagnoses (9.6%). The findings were similar to the primary analysis after adjustment for the indicator (the mental health drugs cluster: HR = 1.54 versus 1.55 for all-cause mortality, SHR = 2.03 versus 2.11 for CVD mortality). The supplementary analysis was carried out to examine whether the availability of information on the variables of interest might lead to potential bias (Additional file 1: Table S9). Participants with missing data tended to have functional impairment, low physical activity, and significant depressive symptoms than complete cases, although most characteristics were similar between the two groups.

## Discussion

Among people with polypharmacy, five high-risk medication patterns—a RAAS inhibitors cluster, a mental health drugs cluster, a CNS drugs cluster, a RAAS inhibitors and antithrombotics cluster, and an antithrombotics cluster—were identified using an agglomerative hierarchical clustering method. Over the 6-year follow-up, the mental health drugs cluster showed increased risk of all-cause mortality (HR = 1.55) and CVD mortality (SHR = 2.11) compared with the CNS drugs cluster, while none of the other medication patterns (single or combined use of RAAS inhibitors and antithrombotics) showed differences in mortality. Apart from medications for mental illness and CVD, the mental health drugs cluster also had a higher prevalence of opioids (33.2% versus 18.6%) and muscle relaxants (21.5% versus 4.4%) than the CNS drugs cluster. These findings suggest that older adults with polypharmacy who take medication for mental disorders (primarily antidepressants), opioids, and muscle relaxants have added risks of all-cause and CVD mortality when their polypharmacy status is positively associated with mortality. The robustness of the main findings was largely confirmed by the sensitivity analyses; the reliability of the significant associations could be assured by the estimated adequate power.

The mechanisms that account for the increased risk of mortality with mental health drugs, opioids, and muscle relaxants among people with polypharmacy may potentially involve drug-drug interactions or comorbidities. Antidepressants that include tricyclic antidepressants, selective serotonin reuptake inhibitors (SSRIs), and serotonin and norepinephrine reuptake inhibitors have shown many pharmacokinetic and pharmacodynamic interactions with other medications, and some of these are of clinical significance, such as serotonin syndrome [42]. For example, antidepressants in combination with fentanyl (long-acting opioids) or lithium (antimanic agents) are likely to promote serotonin syndrome. Older people on antidepressants have also been confirmed to have a higher number of comorbidities; therefore, a higher proportion of people have at least one potential treatment conflict between other conditions (e.g. CVD and arthritis or pain management) and antidepressants [43].

Similarly, major potential drug-drug interactions between opioids and other medications have been reported where opioids are frequently prescribed with antifungal agents, antibiotics, CCBs, antiarrhythmics, SSRIs, or anticonvulsants for chronic pain opioid users [44]. In the mental health drugs cluster, 33.2% were opioid users, and such interactions could have had a major clinical influence. Opioid prescription at discharge from hospital has also been found to be related to the greater illness burden (i.e. higher multimorbidity severity) among hospitalised older people [45]. In addition, a study of breast cancer survivors provided a link between mental disorders and opioid use, implying that this association might be present among older adults as well [46].

There are also concerns about drug-drug interactions with muscle relaxants, including quinine, diazepam, and baclofen [47]. However, there has been no systematic discussion of the drug-drug interactions of muscle relaxants because they include diverse drug classes. Both opioids and muscle relaxants are commonly prescribed for pain management, and they both simultaneously showed the highest prevalence in the mental health drugs cluster. To summarise, the use of antidepressants and opioids may lead to clinically important drug-drug interactions and treatment conflicts with conditions. This situation is likely to be more complicated and unpredictable for older adults with polypharmacy and may account for the increased mortality in the mental health drugs cluster.

Although exclusively long-term medications were considered to define polypharmacy, the medications were taken not only for conditions (e.g. antihyperglycemic agents) but also for symptoms (e.g. pain relief), including over-the-counter medications, as shown in Additional file 1: Table S1. The purpose of this study was to reflect the concurrent medications used in a real-life manner instead of limiting them to prescribed medications, and to identify the patterns of high-risk medications that would affect the risk of mortality. Given the complexity of polypharmacy, medication adherence is another concern because the omission of essential medications may influence subsequent health outcomes (i.e. mortality). Polypharmacy has been found to negatively affect medication adherence among older adults [48, 49], with various barriers to adherence reported that include patient-related factors (e.g. health literacy), drug-related factors (e.g. adverse effects), the patient-provider relationship, difficulties of obtaining medications, and the use of non-prescription medications [48, 50].

### Comparison with existing literature

To our knowledge, this was the first study to investigate the association between high-risk medication patterns and mortality among older adults with polypharmacy; thus, direct comparisons with previous studies are difficult to make.

In the literature, only all-cause mortality has been widely explored, rather than cause-specific mortality. The finding concerning the relationship between mental health drugs and mortality is supported by the literature [16, 51, 52], although some studies have focused on exposure to antipsychotics in schizophrenia patients [51] or older adults with dementia [52]. The finding that opioids are associated with higher mortality is also in line with previous literature [16, 17, 53], including samples of people with chronic non-cancer pain [16, 53] or at least one hospitalisation during the study period [17]. However, some differences between this study and the previous literature can be observed. The use of muscle relaxants was linked to increased mortality in this study, while previous studies have shown a lower risk [16]. Also, this study did not find an association between anticholinergics and mortality as Sevilla-Sanchez et al.’s research [54], whereas the use of anticholinergics has shown a higher risk of mortality in previous studies [13–15]. The difference in the medication classifications used may explain the lack of association in this study. This study adopted 14 high-risk medication categories based on their pharmacological mechanisms (e.g. antidepressants and the remaining anticholinergics), whereas the anticholinergic cognitive burden scale in the literature has included wide-ranging drug classes such as paroxetine (an antidepressant), fentanyl (an opioid), and nifedipine (a CCB) [55].

### Strengths and limitations

This study had several strengths. First, the medication profiles were collected by nurses rather than self-reported by participants, and they were used to verify the self-reported health conditions. This verification and collection process helped to reduce reporting bias. Second, a rigorous definition of polypharmacy was chosen that included medications in long-term use and excluded the temporary use of painkillers. Third, over-the-counter medications for long-term conditions were also included, since some interactions between over-the-counter and prescribed medications might be a concern. Fourth, the study employed a nationally representative sample followed up for 6 years, for whom comprehensive characteristics were available ranging from socio-demographic characteristics to health status. Fifth, a wider range of potential confounders was adjusted for statistically than in previous research, including cognitive function, mobility impairment, lifestyle factors, and depressive symptoms. Lastly, this study used advanced statistical techniques—cluster analysis and survival analysis—to investigate the association between high-risk medications and mortality. The adoption of cluster analysis allows researchers to take concurrent medications into account, which is different from traditional analyses using separate models for each drug class. Concurrent medications are complicated among older adults with polypharmacy and may interact with other medications and further influence mortality. Competing-risks analysis was used for cause-specific mortality to take account of the event of interest and competing events simultaneously, and thus, the estimates should be more accurate [56].

Some limitations of this study should also be acknowledged. Information was collected during the nurse visits on medication type but not on duration, dose, or frequency. These factors are likely to be important in determining whether polypharmacy has adverse effects. For example, specific medications at high doses may impact mortality markedly, as in the case of opioids that have been identified as particularly problematic in the polypharmacy guidance from the Scottish government [23]. Furthermore, the collection of medication information was made at a single time point, and the medicines may have changed over the follow-up period. Although this study employed a rigorous definition of long-term medications and broad medication categories that included one or more drug classes to minimise the bias caused by the change in the medications, unmeasured time bias could not be avoided [57]. Also, the effectiveness of the medications could not be assessed due to the nature of one-time collection and a lack of information. Moreover, the exclusion (i.e. cancer patients) and attrition of the study sample might have limited the representativeness of this study, even though the supplementary analysis suggested this was not the case. Lastly, the lack of a significant association between cause-specific mortality and the medication patterns might be due to low statistical power, attributable to the small number of deaths.

### Implications for clinical practice

NHS England’s medication review service is in transition at the moment, moving away from the medicines use reviews (MURs) commissioned from community pharmacies and towards enhanced ‘structured medication reviews’ carried out by clinical pharmacists [58]. In addition to people with polypharmacy and heightened polypharmacy who are advised to have medication reviews according to NICE [22], and NHS England [3] and Scottish government guidance [23], respectively, this study provides more information on high-risk medications that contribute to increased mortality among older people with polypharmacy. The results of this study somewhat confirm the effectiveness of MURs that were introduced in 2005 and ended in the 2020–2021 financial year [59]. One of the target groups for MURs is patients who take high-risk medicines, including NSAIDs, anticoagulants, antiplatelets, and diuretics [59], and none of them showed an association with increased mortality in this study. Furthermore, some medication use has been highlighted in the polypharmacy guidance issued by the NHS England and NHS Scotland. The service model of structured medication reviews proposed by the NHS England includes addictive pain management drugs (e.g., opioids) [3], while the Scottish government has set up a long list of high-risk medications [23]. In the list of high-risk medications, no mental health drugs or muscle relaxants are discussed except for lithium, but opioids at high doses and in long-term use are emphasised. This study supports the inclusion of opioids in the current guidance, but it also suggests that older adults with polypharmacy who take mental illness medications and muscle relaxants are prone to suffer from adverse outcomes and therefore may need more attention. These results are expected to provide more evidence to improve the service model of structured medication reviews, contributing to early intervention for older adults with polypharmacy and on specific medications. Early intervention in medication use, such as the close monitoring of specific medications and regular medication reviews, would ensure treatment appropriateness and medication optimisation, reduce polypharmacy-related problems such as adverse effects, drug-drug interactions, and redundant medications, and potentially bring clinical benefits to older people with polypharmacy.

## Conclusion

Among older people with polypharmacy in England, the concurrent use of mental health drugs (primarily antidepressants), opioids, and muscle relaxants was found to increase the risk of all-cause and CVD mortality, compared with other medication patterns. This study supports that addictive pain management medications should be included in the structured medication reviews of older adults with polypharmacy, but it also suggests that the prescription of mental health medications and muscle relaxants may need more attention. The reinforcement of structured medication reviews would contribute to early intervention in medication use, and it may help to reduce polypharmacy-related problems and bring clinical benefits to older people.

## Supplementary Information

**Additional file 1: Table S1.** Medication categories in long-term use, ELSA 2012. **Table S2.** Number of missing values for variables in the model (N = 1705), ELSA 2012. **Table S3.** Fourteen high-risk medication categories, ELSA 2012. **Table S4.** Self-reported and verified long-term conditions, ELSA 2012. **Table S5.** Comparison between four-cluster and five-cluster for the prevalence of 14 high-risk medication categories and model fit statistics. **Table S6.** Power calculations for survival analysis. **Table S7.** Additional baseline characteristics of people with polypharmacy (N = 1356) by cluster, ELSA 2012. **Table S8.** Sensitivity analyses of the associations between medication patterns and mortality in England in 2012 − 18. **Table S9.** Comparison of characteristics between the sample with and without missing data. **Figure S1.** Flow chart of samples for cluster and survival analyses. **Figure S2.** Dendrogram of cluster analysis in people with polypharmacy.

## Data Availability

The data are available through the UK Data Service.

## Abbreviations

ACEI: Angiotensin-converting enzyme inhibitor
ADLs: Activities of daily living
BMI: Body mass index
BZD: Benzodiazepine
CCB: Calcium channel blocker
CHD: Coronary heart disease
CI: Confidence interval
CIF: Cumulative incidence function
CNS: Central nervous system
CVD: Cardiovascular disease
ELSA: English Longitudinal Study of Ageing
HR: Hazard ratio
IADLs: Instrumental activities of daily living
MUR: Medicines use review
NHS: National Health Service
NICE: National Institute for Health and Care Excellence
NSAID: Non-steroidal anti-inflammatory drug
RAAS: Renin-angiotensin-aldosterone system
SA1: The first sensitivity analysis
SA2: The second sensitivity analysis
SA3: The third sensitivity analysis
SA4: The fourth sensitivity analysis
SA5: The fifth sensitivity analysis
SHR: Subdistribution hazard ratio
SSRI: Selective serotonin reuptake inhibitor
START: Screening tool to alert to right treatment
STOPP: Screening tool of older people’s prescriptions
