# Supplementary material for: The impact of high-risk medications on mortality risk among older adults with polypharmacy: evidence from the English Longitudinal Study of Ageing
Source: BMC Med. 2021 Dec 16;19:321. doi: 10.1186/s12916-021-02192-1 (PMC8675465; doi:10.1186/s12916-021-02192-1)
Supplement: Supplementary file 1 — Additional file 1: Table S1. Medication categories in long-term use, ELSA 2012. Table S2. Number of missing values for variables in the model (N = 1705), ELSA 2012. Table S3. Fourteen high-risk medication categories, ELSA 2012. Table S4. Self-reported and verified long-term conditions, ELSA 2012. Table S5. Comparison between four-cluster and five-cluster for the prevalence of 14 high-risk medication categories and model fit statistics. Table S6. Power calculations for survival analysis. Table S7. Additional baseline characteristics of people with polypharmacy (N = 1356) by cluster, ELSA 2012. Table S8. Sensitivity analyses of the associations between medication patterns and mortality in England in 2012 − 18. Table S9. Comparison of characteristics between the sample with and without missing data. Figure S1. Flow chart of samples for cluster and survival analyses. Figure S2. Dendrogram of cluster analysis in people with polypharmacy. [file 12916_2021_2192_MOESM1_ESM.docx]

**Additional file 1**

Table S1 Medication categories in long-term use, ELSA 2012

| Long-term condition | Medication category |  |
| --- | --- | --- |
| Diabetes | Insulin, sulphonylureas (SUs), biguanides (metformin), dipeptidyl peptidase-4 (DPP-4) inhibitors, meglitinides, thiazolidinediones (TZDs), glucagon-like peptide-1 (GLP-1) agonists, α-glucosidase inhibitor, and sodium-glucose cotransporter-2 (SGLT-2) inhibitors |  |
| Diabetic neuropathy | Codeine and carbamazepine |  |
| CVDs | Digoxin, diuretics (thiazide, loop, potassium-sparing and combinations), antiarrhythmics, β blockers, α2 agonists, α1 blockers, calcium channel blockers (CCBs), renin-angiotensin-aldosterone system (RAAS) inhibitors (angiotensin-converting-enzyme inhibitors (ACEIs), angiotensin II receptor blockers (ARBs), and renin inhibitors), vasodilators, and antithrombotics (anticoagulants and anti-platelets) |  |
| Hyperlipidaemia | Statins, fibrates, niacin, bile acid sequestrants, omega-3 fatty acids, cholesterol absorption inhibitors, and microsomal triglyceride transfer protein inhibitors |  |
| Hyperuricemia (including gout) | Allopurinol and non-steroidal anti-inflammatory drugs (NSAIDs) (for acute attack) |  |
| Lung disease (including inhalers) | Steroids (oral and inhaled), β2 agonists (including long-acting), anticholinergics, theophylline, aminophylline, combinations (e.g. ipratropium plus salbutamol), mast cell stabilisers, and leukotriene receptor antagonists |  |
| Bone disease | Bisphosphonates |  |
| Psychiatric conditions | Tricyclic antidepressants (TCAs), selective serotonin reuptake inhibitors (SSRIs), serotonin and norepinephrine reuptake inhibitors (SNRIs), typical antipsychotics, atypical antipsychotics, antimanic agents, and anxiolytics |  |
| Epilepsy (seizure) | Anticonvulsants, benzodiazepines (BZDs), and phenytoin |  |
| Parkinson’s disease | Carbidopa-levodopa and anticholinergic (procyclidine) |  |
| Dementia (including Alzheimer’s disease) | Acetylcholinesterase inhibitor (donepezil) and glutamate receptor antagonist (memantine) |  |
| Inflammatory bowel disease | Metronidazole, sulfasalazine, mesalazine, steroids, and immunosuppressants |  |
| Autoimmune disease (rheumatic disease, myasthenia gravis) | Steroids and immunosuppressants |  |
| Cancer | Immunosuppressants, steroids, oral chemotherapy, methotrexate, hormones, selective oestrogen receptor modulators, aromatase inhibitors, and gonadotropin-releasing hormone agonists |  |
| Long-term symptom | Medication category | |
| Hormone therapy | Thyroxine (levothyroxine), steroids, sex hormones (including patches), gonadotropin-releasing hormone agonists, and contraceptives (not including emergency contraceptives) | |
| Treatment for substance dependence (including alcohol, opioids, and smoking) | Including all forms of nicotine replacement therapy | |
| Sedative (hypnotic) | BZD and non-BZD derivatives | |
| Tremor | Propranolol | |
| Symptom relief for pain, inflammation, and rheumatic disease | NSAIDs (including aspirin) | |
| Pain relief | Opioid derivatives, and drugs for trigeminal neuralgia and migraine (including headache) | |
| Peptic ulcers and gastroesophageal reflux disease | H_2_-receptor blockers, prostaglandin E1 analogue, and proton-pump inhibitors | |
| Supplements for people with bone disease | Calcium products | |
| Attention deficit hyperactivity disorder | Methylphenidate | |
| Tourette’s syndrome | Sulpiride | |
| Benign prostatic hyperplasia | 5α reductase inhibitors and α1 blockers | |
| Urinary incontinence | Anticholinergics (oxybutynin) and antimuscarinics (trospium chloride and solifenacin) | |
| Urine alkalinisation, ureteric colic | Sodium bicarbonate and diclofenac | |
| Thyrotoxicosis | Propranolol | |
| Nocturnal cramps | Muscle relaxants (quinine, diazepam, and baclofen) | |
| Dry mouth | Pilocarpine | |
| Sputum viscosity | Carbocisteine | |

Table S2 Number of missing values for variables in the model (N=1705), ELSA 2012

|  | N (%) |
| --- | --- |
| Age | 0 (0) |
| Gender | 0 (0) |
| Live with a partner | 0 (0) |
| Diabetes mellitus | 0 (0) |
| CHD | 0 (0) |
| Stroke | 0 (0) |
| Lung disease (including asthma) | 0 (0) |
| Number of conditions | 0 (0) |
| Functional impairment | 0 (0) |
| Mobility difficulty | 0 (0) |
| Obesity (BMI and waist circumference) | 185 (10.9) |
| Current smoker | 0 (0) |
| Sleep < 7 or > 9 hours | 2 (0.1) |
| Low physical activity | 1 (0.1) |
| Depressive symptoms 4+ | 32 (1.9) |
| Total wealth (quintile) | 135 (7.9) |
| Parkinson’s disease | 0 (0) |
| Dementia (including Alzheimer’s disease) | 0 (0) |
| Cognitive function | 0 (0) |

Table S3 Fourteen high-risk medication categories, ELSA 2012

| **Category** | **Medication** |
| --- | --- |
| Benzodiazepines (BZDs) | Sedatives: BZD and non-BZD derivatives |
|  | Anxiolytic: lorazepam and diazepam |
|  | Antiepileptic: lorazepam and diazepam |
| Antipsychotics | Atypical antipsychotics |
|  | Typical antipsychotics |
|  | Tourette’s syndrome: sulpiride |
| Antidepressants | Tricyclic antidepressants (TCAs) |
|  | Selective serotonin reuptake inhibitors (SSRIs) |
|  | Serotonin and norepinephrine reuptake inhibitors (SNRIs) |
| Antimanic agents | Lithium and carbamazepine |
| Calcium channel blockers (CCBs) | Both dihydropyridines and non-dihydropyridines |
| Diuretics | Thiazide-like diuretics |
|  | Loop diuretics |
|  | Potassium-sparing diuretics |
|  | Combination: potassium-sparing + thiazide/loop |
| Renin-angiotensin-aldosterone system (RAAS) inhibitors | Angiotensin-converting-enzyme inhibitors (ACEIs) |
|  | Angiotensin II receptor blockers (ARBs) |
|  | Renin inhibitors |
| Opioids for pain relief | Opioid derivatives |
| Muscle relaxants | Quinine, diazepam, and baclofen |
| Non-steroidal anti-inflammatory drugs (NSAIDs)^§^ | Including aspirin |
| Antithrombotics | Anticoagulants |
|  | Anti-platelets |
| Steroids^§^ | Hormone therapy: hydrocortisone |
|  | Hormone therapy: prednisolone |
|  | Pulmonary: prednisolone |
|  | Pulmonary: hydrocortisone |
|  | Inflammatory bowel disease: prednisolone and hydrocortisone |
|  | Rheumatic disease: prednisolone |
| Anticholinergics^#^ | Urinary incontinence: oxybutynin, trospium chloride, and solifenacin |
|  | Smoking cessation aid  Nicotine replacement therapy (all forms) |
|  | Parkinson’s disease: procyclidine |
| Other central nervous system (CNS) drugs | Migraine/headache: analgesics |
|  | Epilepsy: anticonvulsants |
|  | Trigeminal neuralgia: anticonvulsants |
|  | Parkinson’s disease: carbidopa-levodopa |
|  | Alzheimer's disease:  Acetylcholinesterase inhibitor: donepezil  Glutamate receptor antagonist: memantine |
|  | Dry mouth: pilocarpine |
|  | Attention deficit hyperactivity disorder: methylphenidate |
|  | Alcohol dependence |

^§^ Oral form only.

^#^ Remaining anticholinergics not included in other medication categories.

**Table S4 Self-reported and verified long-term conditions, ELSA 2012**

| Self-reported diagnosis | N (%) | Verified diagnosis | N (%) |
| --- | --- | --- | --- |
| Diabetes | 406 (29.9) | Diabetes^#^ | 473 (34.9) |
| Lung disease | 136 (10.0) | Lung disease (including asthma) | 423 (31.2) |
| Asthma | 281 (20.7) |  |  |
| Osteoporosis | 207 (15.3) | Bone disease^#^ | 254 (18.7) |
| Parkinson’s disease | 10 (0.7) | Parkinson’s disease | 23 (1.7) |
| Alzheimer’s disease | 6 (0.4) | Dementia (including Alzheimer’s disease) | 27 (2.0) |
| Dementia | 22 (1.6) |  |  |
| Psychiatric conditions | 193 (14.2) | Psychiatric conditions | 381 (28.1) |
| Hyperlipidaemia | 812 (59.9) |  |  |
| Hypertension | 954 (70.4) |  |  |
| Stroke | 166 (12.2) |  |  |
| CHD | 396 (29.2) |  |  |
| Other heart problems | 399 (29.4) |  |  |
| Arthritis | 798 (58.9) |  |  |
| Any 1 of 4 eye diseases | 685 (50.5) |  |  |
| Diagnosis defined by specific treatments | | | |
| Hyperuricemia (including gout) | 83 (6.1) |  |  |
| Epilepsy | 66 (4.9) |  |  |
| Inflammatory bowel disease | 14 (1.0) |  |  |

^#^ Diabetes included diagnosed and undiagnosed cases. Bone disease included osteoporosis, Paget’s disease, and heterotopic ossification.

**Table S5 Comparison between four-cluster and five-cluster for the prevalence of 14 high-risk medication categories and model fit statistics**

| Medication category % (N) | Cluster 1 (N=194) | Cluster 2 (N=298) | Cluster 3 (N=387) | Cluster 4 (N=477) | Cluster 4^*^ (N=352) | Cluster 5^*^ (N=125) |
| --- | --- | --- | --- | --- | --- | --- |
| BZDs | 4.1 (8) | 10.4 (31) | 9.3 (36) | 0.2 (1) | 0.3 (1) | 0 (0) |
| Antipsychotics | 0.5 (1) | 2.4 (7) | 1.6 (6) | 0.2 (1) | 0.3 (1) | 0 (0) |
| Antidepressants | 0 (0) | 64.1 (191) | 30.0 (116) | 0 (0) | 0 (0) | 0 (0) |
| Antimanics | 0 (0) | 0.7 (2) | 0.5 (2) | 0 (0) | 0 (0) | 0 (0) |
| CCBs | 49.0 (95) | 39.3 (117) | 26.6 (103) | 40.9 (195) | 40.1 (141) | 43.2 (54) |
| Diuretics | 58.3 (113) | 42.6 (127) | 23.8 (92) | 42.1 (201) | 42.9 (151) | 40.0 (50) |
| RAAS inhibitors | 83.5 (162) | 66.8 (199) | 33.6 (130) | 73.4 (350) | **99.4 (350)** | **0 (0)** |
| Opioids | 0 (0) | 33.2 (99) | 18.6 (72) | 0.8 (4) | 0.3 (1) | 2.4 (3) |
| Muscle relaxants | 0 (0) | 21.5 (64) | 4.4 (17) | 0.2 (1) | 0 (0) | 0.8 (1) |
| NSAIDs | 0 (0) | 10.4 (31) | 30.2 (117) | 2.5 (12) | 0 (0) | 9.6 (12) |
| Antithrombotics | 0 (0) | 64.8 (193) | 25.8 (100) | 100.0 (477) | **100.0 (352)** | **100.0 (125)** |
| Steroids | 0 (0) | 2.4 (7) | 19.1 (74) | 0 (0) | 0 (0) | 0 (0) |
| Anticholinergics | 0 (0) | 2.7 (8) | 15.0 (58) | 1.9 (9) | 2.6 (9) | 0 (0) |
| Other CNS drugs | 0.5 (1) | 3.0 (9) | 32.3 (125) | 0.2 (1) | 0 (0) | 0.8 (1) |
| Model fit |  |  |  | Four-cluster |  | Five-cluster |
| Log likelihood |  |  |  | -1521.67 |  | -1521.29 |
| AIC |  |  |  | 3095.34 |  | 3096.574 |
| BIC |  |  |  | 3230.859 |  | 3237.306 |

^*^ The fourth cluster was split into two clusters to become the five clusters.

**Table S6 Power calculations for survival analysis**

|  | **Cluster 1**  **RAAS inhibitors** | **Cluster 2**  **Mental health drugs** | **Cluster 3**  **CNS drugs** | **Cluster 4**  **RAAS inhibitors + antithrombotics** | **Cluster 5**  **Antithrombotics** |
| --- | --- | --- | --- | --- | --- |
|  | (N=194) | (N=298) | (N=387) | (N=352) | (N=125) |
| **All-cause mortality** % (N) | 15.5 (30) | 22.2 (66) | 12.9 (50) | 16.8 (59) | 24.0 (30) |
| Estimated power | 13.6% | 89.3% | Ref. | 32.0% | 81.6% |
| **CVD mortality** % (N) | 4.6 (9) | 10.4 (31) | 4.1 (16) | 8.5 (30) | 8.0 (10) |
| Estimated power | 5.9% | 89.8% | Ref. | 70.1% | 37.8% |
| **Non-CVD mortality** % (N) | 10.8 (21) | 11.7 (35) | 8.8 (34) | 8.2 (29) | 16.0 (20) |
| Estimated power | 12.0% | 23.8% | Ref. | 6.0% | 59.0% |

Table S7 Additional baseline characteristics^†^ of people with polypharmacy (N=1356) by cluster, ELSA 2012

|  | **Cluster 1**  **RAAS inhibitors** | **Cluster 2**  **Mental health drugs** | **Cluster 3**  **CNS drugs** | **Cluster 4**  **RAAS inhibitors + antithrombotics** | **Cluster 5**  **Antithrombotics** |
| --- | --- | --- | --- | --- | --- |
|  | **(N=194)**  **% (N)** | **(N=298)**  **% (N)** | **(N=387)**  **% (N)** | **(N=352)**  **% (N)** | **(N=125)**  **% (N)** |
| Total wealth |  |  |  |  |  |
| 1 (lowest) | 26.3 (51) | 34.6 (103) | 29.7 (115) | 24.2 (85) | 30.4 (38) |
| 2 | 22.2 (43) | 24.8 (74) | 23.5 (91) | 21.0 (74) | 23.2 (29) |
| 3 | 22.2 (43) | 19.5 (58) | 17.3 (67) | 21.6 (76) | 18.4 (23) |
| 4 | 16.0 (31) | 13.1 (39) | 19.4 (75) | 19.3 (68) | 20.0 (25) |
| 5 (highest) | 13.4 (26) | 8.1 (24) | 10.1 (39) | 13.9 (49) | 8.0 (10) |
| Parkinson’s disease | 0 | 0.7 (2) | 5.2 (20) | 0 | 0.8 (1) |
| Dementia (including Alzheimer’s disease) | 1.0 (2) | 3.4 (10) | 3.4 (13) | 0.3 (1) | 0.8 (1) |
| Cognitive function mean (SD) | 10.0 (3.2) | 9.4 (3.6) | 9.7 (3.7) | 9.8 (3.2) | 9.3 (3.7) |

^†^ Including two variables (total wealth and cognitive function) with similar distributions across the five clusters and two conditions (Parkinson’s disease and dementia (including Alzheimer’s disease)) with low prevalence rates.

Table S8 Sensitivity analyses of the associations between medication patterns* and mortality in England in 2012−18

|  | **Cluster 1**  **RAAS inhibitors** | | **Cluster 2**  **Mental health drugs** | | **Cluster 4**  **RAAS inhibitors + antithrombotics** | | **Cluster 5**  **Antithrombotics** | |
| --- | --- | --- | --- | --- | --- | --- | --- | --- |
| All-cause mortality, N=1356 (235 deaths) | HR (95% CIs) | P | HR (95% CIs) | P | HR (95% CIs) | P | HR (95% CIs) | P |
| Main model | 1.56 (0.97, 2.50) | 0.064 | 1.55 (1.05, 2.28) | **0.028** | 1.17 (0.78, 1.76) | 0.454 | 1.43 (0.89, 2.30) | 0.140 |
| SA1. Main model with separate CVD conditions**^║^** | 1.56 (0.97, 2.51) | 0.068 | 1.53 (1.03, 2.26) | **0.033** | 1.12 (0.74, 1.70) | 0.597 | 1.41 (0.88, 2.27) | 0.156 |
| SA2. Main model with separate psychiatric conditions | 1.57 (0.97, 2.52) | 0.065 | 1.54 (1.02, 2.32) | **0.041** | 1.17 (0.78, 1.77) | 0.450 | 1.43 (0.89, 2.31) | 0.140 |
| SA3. Main model + alcohol consumption^#^ | 1.56 (0.93, 2.64) | 0.094 | 1.70 (1.10, 2.63) | **0.017** | 1.13 (0.72, 1.78) | 0.582 | 1.37 (0.81, 2.33) | 0.237 |
| SA4. Main model + taking medications but without diagnoses^§^ | 1.56 (0.97, 2.50) | 0.064 | 1.54 (1.04, 2.29) | **0.030** | 1.17 (0.78, 1.75) | 0.455 | 1.43 (0.89, 2.30) | 0.142 |
| CVD mortality, N=1356 (96 deaths) | SHR (95% CIs) | P | SHR (95% CIs) | P | SHR (95% CIs) | P | SHR (95% CIs) | P |
| Main model | 1.26 (0.55, 2.91) | 0.583 | 2.11 (1.10, 4.05) | **0.024** | 1.49 (0.76, 2.89) | 0.243 | 1.17 (0.50, 2.76) | 0.721 |
| SA1. Main model with separate CVD conditions**^║^** | 1.27 (0.55, 2.97) | 0.578 | 2.10 (1.09, 4.04) | **0.027** | 1.43 (0.72, 2.84) | 0.301 | 1.16 (0.49, 2.73) | 0.739 |
| SA2. Main model with separate psychiatric conditions | 1.25 (0.54, 2.91) | 0.598 | 2.13 (1.07, 4.27) | **0.032** | 1.48 (0.75, 2.92) | 0.262 | 1.16 (0.49, 2.75) | 0.730 |
| SA3. Main model + alcohol consumption^#^ | 1.95 (0.76, 5.04) | 0.166 | 3.04 (1.42, 6.52) | **0.004** | 1.83 (0.82, 4.08) | 0.139 | 1.36 (0.49, 3.72) | 0.554 |
| SA4. Main model + taking medications but without diagnoses^§^ | 1.27 (0.55, 2.92) | 0.571 | 2.03 (1.04, 3.95) | **0.038** | 1.49 (0.77, 2.87) | 0.239 | 1.14 (0.48, 2.70) | 0.762 |
| Non-CVD mortality, N=1356 (139 deaths) | SHR (95% CIs) | P | SHR (95% CIs) | P | SHR (95% CIs) | P | SHR (95% CIs) | P |
| Main model | 1.48 (0.80, 2.73) | 0.214 | 1.18 (0.72, 1.94) | 0.518 | 0.93 (0.55, 1.57) | 0.774 | 1.49 (0.82, 2.70) | 0.189 |
| SA1. Main model with separate CVD conditions**^║^** | 1.47 (0.80, 2.71) | 0.218 | 1.17 (0.71, 1.92) | 0.541 | 0.90 (0.52, 1.53) | 0.691 | 1.47 (0.82, 2.66) | 0.196 |
| SA2. Main model with separate psychiatric conditions | 1.50 (0.80, 2.79) | 0.206 | 1.15 (0.68, 1.96) | 0.598 | 0.94 (0.55, 1.59) | 0.812 | 1.51 (0.83, 2.74) | 0.179 |
| SA3. Main model + alcohol consumption^#^ | 1.36 (0.72, 2.58) | 0.344 | 1.03 (0.59, 1.82) | 0.914 | 0.77 (0.43, 1.38) | 0.383 | 1.28 (0.65, 2.53) | 0.474 |
| SA4. Main model + taking medications but without diagnoses^§^ | 1.47 (0.80, 2.72) | 0.218 | 1.19 (0.72, 1.98) | 0.493 | 0.93 (0.55, 1.57) | 0.775 | 1.50 (0.82, 2.73) | 0.186 |

^*^ Cluster 3 CNS drugs (N=387) as the reference group.

**^║^** Four cardiovascular-related diagnoses − CHD, stroke, hypertension, and other heart problems − were adjusted separately instead of combining some diagnoses into an illness count.

^#^ Reduced N=1221 (195 all-cause deaths; 82 CVD deaths; 113 non-CVD deaths).

^§^ A small proportion of people who took medications but did not report relevant diagnoses.

Table S9 Comparison of characteristics between the sample with and without missing data

|  | **Complete sample plus those with missing data**  **(N=1705)** | **Complete sample**    **(N=1356)** |
| --- | --- | --- |
| Age (years) mean (SD) | 71.8 (9.1) | 71.9 (8.7) |
| Women | 54.3 (925) | 54.9 (745) |
| Total wealth (N=1570^*^) |  |  |
| 1 (lowest) | 29.7 (466) | 28.9 (392) |
| 2 | 22.9 (359) | 22.9 (311) |
| 3 | 19.4 (304) | 19.7 (267) |
| 4 | 17.2 (270) | 17.6 (238) |
| 5 (highest) | 10.9 (171) | 10.9 (148) |
| Living with a partner | 59.9 (1022) | 62.6 (849) |
| Diabetes mellitus | 36.1 (615) | 34.9 (473) |
| CHD | 30.3 (517) | 29.2 (396) |
| Stroke | 13.3 (226) | 12.2 (166) |
| Lung disease (including asthma) | 32.4 (553) | 31.2 (423) |
| Parkinson’s disease | 2.1 (35) | 1.7 (23) |
| Dementia (including Alzheimer’s disease) | 2.3 (39) | 2.0 (27) |
| Number of conditions^#^ median (IQR) | 3 (2) | 3 (2) |
| Functional impairment | 45.6 (778) | 40.4 (548) |
| Mobility difficulty | 81.8 (1395) | 79.5 (1078) |
| Obesity (N=1520^*^) |  |  |
| High BMI and waist circumference | 44.5 (677) | 43.1 (585) |
| Either high BMI or high waist circumference | 25.5 (388) | 25.9 (351) |
| Current smoker | 13.7 (234) | 13.0 (176) |
| Sleep < 7 or > 9 hours (N=1703^*^) | 46.9 (799) | 45.9 (622) |
| Low physical activity (N=1704^*^) | 43.9 (748) | 38.8 (526) |
| Depressive symptoms 4+ (N=1673^*^) | 22.4 (375) | 18.9 (256) |
| Cognitive function mean (SD) | 9.3 (3.6) | 9.7 (3.5) |

^*^ The available data among 1705 individuals. The analytical sample of 1356 individuals was complete cases.

Figure S1 Flow chart of samples for cluster and survival analyses

Polypharmacy:

1705 participants

(1) 231

(2) 396

(3) 495

(4) 433

(5) 150

Cluster analysis

(1) 194

(2) 298

(3) 387

(4) 352

(5) 125

Exclude people who had incomplete data (N=328) and less than 1-year follow-up (N=21), resulting in 1356 participants for survival analysis

9169 core member interviews

7730 core member nurse visits

7227 participants

Exclude people who had incomplete data (N = 4)^*^ or who had been diagnosed with cancer^#^

(N = 480) or taking cancer medications^†^ (N = 19)

No polypharmacy:

5522 participants

^*^ People without information on diabetes diagnosis (N = 1), physical activity and functioning (N = 2) and follow-up time (N = 1).

^#^ Including cancer and malignant blood disorders self-reported by participants.

^†^ Referring to hormone therapy that is primarily for cancers.

Figure S2 Dendrogram of cluster analysis in people with polypharmacy

396

495

433
